# Severe root resorption resulting from orthodontic treatment: Prevalence and risk factors

**DOI:** 10.1590/2176-9451.20.1.052-058.oar

**Published:** 2015

**Authors:** Caroline Pelagio Raick Maués, Rizomar Ramos do Nascimento, Oswaldo de Vasconcellos Vilella

**Affiliations:** 1 DDS in Dentistry, Fluminense Federal University (UFF); 2 Specialist in Orthodontics, UFF; 3 Postgraduate program in Orthodontics, UFF

**Keywords:** Epidemiology, Root resorption, Orthodontics

## Abstract

**OBJECTIVE:**

To assess the prevalence of severe external root resorption and its potential risk factors resulting from orthodontic treatment.

**METHODS:**

A randomly selected sample was used. It comprised conventional periapical radiographs taken in the same radiology center for maxillary and mandibular incisors before and after active orthodontic treatment of 129 patients, males and females, treated by means of the Standard Edgewise technique. Two examiners measured and defined root resorption according to the index proposed by Levander et al. The degree of external apical root resorption was registered defining resorption in four degrees of severity. To assess intra and inter-rater reproducibility, kappa coefficient was used. Chi-square test was used to assess the relationship between the amount of root resorption and patient's sex, dental arch (maxillary or mandibular), treatment with or without extractions, treatment duration, root apex stage (open or closed), root shape, as well as overjet and overbite at treatment onset.

**RESULTS:**

Maxillary central incisors had the highest percentage of severe root resorption, followed by maxillary lateral incisors and mandibular lateral incisors. Out of 959 teeth, 28 (2.9%) presented severe root resorption. The following risk factors were observed: anterior maxillary teeth, overjet greater than or equal to 5 mm at treatment onset, treatment with extractions, prolonged therapy, and degree of apex formation at treatment onset.

**CONCLUSION:**

This study showed that care must be taken in orthodontic treatment involving extractions, great retraction of maxillary incisors, prolonged therapy, and/or completely formed apex at orthodontic treatment onset.

## Abstract (pt)

**OBJETIVO:**

avaliar a prevalência de reabsorções radiculares externas severas e identificar prováveis fatores de risco decorrentes do tratamento ortodôntico.

**MÉTODOS:**

utilizou-se uma amostra selecionada aleatoriamente, composta de radiografias periapicais de incisivos superiores e inferiores, obtidas no mesmo centro radiológico, de pré- e pós-tratamento ortodôntico ativo, de 129 pacientes, de ambos os sexos, tratados por meio da técnica Edgewise Standard. Dois examinadores mensuraram e definiram a reabsorção radicular de acordo com índice proposto por Levander et al., e o grau de reabsorção foi registrado, definindo a reabsorção em quatro graus de severidade. Para avaliar a reprodutibilidade intra- e interexaminadores, adotou-se o índice de coeficiente *kappa* ponderado. O teste chi-quadrado (χ^2^) foi adotado para avaliar a relação entre a quantidade de reabsorção radicular e o sexo dos pacientes, arcada dentária (superior ou inferior), tratamentos com ou sem extrações, duração do tratamento, forma radicular, estágio do ápice radicular (aberto ou fechado), *overjet* e *overbite* no início do tratamento.

**RESULTADOS:**

os incisivos centrais superiores apresentaram a maior porcentagem de reabsorção radicular severa, seguidos dos incisivos laterais superiores e dos incisivos laterais inferiores. Entre 959 dentes avaliados, 28 (2,9%) apresentaram reabsorção radicular severa. Os fatores de risco relacionados foram: dentes localizados na região anterossuperior, *overjet* maior ou igual a 5mm ao início do tratamento, tratamentos envolvendo extrações dentárias, tempo prolongado de terapia e formação radicular completa à época do início do tratamento ortodôntico.

**CONCLUSÃO:**

o estudo demonstrou que cuidados devem ser tomados em tratamentos ortodônticos envolvendo extrações, com grande retração de incisivos superiores, tratamentos prolongados e/ou ápice radicular completamente formado no início da terapia ortodôntica.

## INTRODUCTION

External apical root resorption (EARR) is an undesirable side effect commonly associated with orthodontically induced tooth movement.^1^^-^ 6 As it is considered a borderline phenomenon between cost-benefit and iatrogenesis, such resorptions gain importance not only due to being highly frequent, with potential biological damage to the patient, but also due to potential legal implications in daily orthodontic practice.

Root shortening results from a combination of complex biological activities in the region of the periodontal ligament, which will interact with force exerted during orthodontic treatment.^7^ Factors such as dental trauma prior to orthodontic treatment, bone density and morphology, shape of teeth roots,^5^^,^ 6 ^,^ 8 patient's age at orthodontic treatment onset,^9^ treatment duration,^5^^,^ 6 ^,^ 8 ^,^ 10 as well as orthodontic mechanics and magnitude of force^2^^,^ 10 ^-^ 15 have been reported as significant for the occurrence of EARR.

Lateral cephalograms associated with panoramic radiograph or complete periapical radiographs are routinely requested for pretreatment planning. Studies highlight better precision of periapical radiograph when compared to panoramic radiograph when determining the magnitude of root resorption, due to lower distortion and accuracy of fine details. Therefore, an increasing number of professionals request complete periapical examination for treatment of adult orthodontic patients.^16^

The aim of this retrospective study was to determine, by means of periapical radiographs, the prevalence of severe EARR (exceeding 1/3 of the original root length) and its relationship with orthodontic treatment variables in patients treated with Edgewise Standard technique. It also assessed potential risk factors. 

## MATERIAL AND METHODS

The present study was submitted to Fluminense Federal University (UFF) Institutional Review Board (protocol #188780) and performed in accordance to its norms.

A randomly selected sample was used. It comprised conventional periapical radiographs taken in the same radiology center for all incisors of 129 patients (males and females) before and after active orthodontic treatment. Patients were treated by means of the Standard Edgewise technique in the last fifteen years at the Orthodontics Department of Fluminense Federal University (UFF). As inclusion criteria, only patients presenting periapical radiographs pre and post-treatment, and those who had completed orthodontic treatment were selected. Exclusion criteria excluded teeth with periapical lesions, history of dental trauma or endodontic treatment, patients with severe crowding in which overlap hindered visualization of roots and subsequent measurements. Low-quality radiographs were also eliminated.

All subjects were treated with conventional metallic non pre-adjusted appliances (Edgewise Standard) with 0.022 x 0.028-in bracket slots, and followed a predetermined archwire sequence during levelling and alignment: For initial leveling, 0.014-in and 0.016-in nickel-titanium (NiTi) archwires were selected, followed by 0.017 × 0.025-in, 0.019 × 0.025-in nickel-titanium (NiTi), and 0.019 × 0.025-in stainless-steel archwires. In cases involving extractions, straight 0.019 × 0.025-in stainless-steel archwires with "T" loops were used to close extraction spaces. No temporary skeletal anchorage devices were used in the selected sample.

**Figure 1 -:**
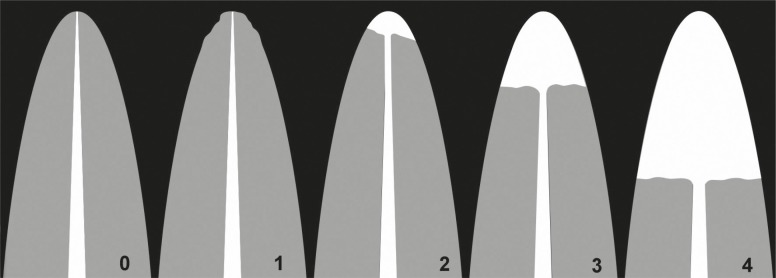
Degrees of external root resorption based on Levander et al9 adding (zero) degree in order to point out unaltered root apex.

Due to applicability and broad acceptance, the index proposed by Malmgren et al^17^ was used to assess the degree of root changes yielded in this study. Zero degree was added to this index, as proposed by Levander et al,^9^ in order to point out unaltered teeth in the root apex (Fig 1).

Tooth length was measured as the distance from the root apex tip to the midpoint of the incisal edge. A digital caliper (Lee Tools, Brazil) with an accuracy of ±0.02 mm and reproducibility of ±0.01 mm was used following the long axis of the tooth. Root contour of maxillary and mandibular incisors assessed before and after treatment were compared, positioning the long axis of the tooth/root parallel to the index image. The degree of EARR was assessed according to the index proposed, using a 0-4 scale of severity, as follows:

» Score 0: Absence of changes in the root apex;
» Score 1: Irregular root contour;
» Score 2: EARR of less than 2 mm;
» Score 3: EARR from 2 mm to one-third of the original root length;
» Score 4: EARR exceeding one-third of the original root length.

Evaluations were carried out by two observers using an x-ray viewer with standard light intensity, equipped with a 5-x magnification loop (Cristófoli Equipamentos de Biossegurança Ltda., Campo Mourão, Paraná, Brazil). After a 15-day interval, measurements were reassessed by the observers using periapical radiographs of 20 patients (160 teeth)randomly selected before and after orthodontic treatment.

A total of 1,032 teeth were evaluated; out of which 73 were excluded, thereby totaling 959 teeth. The prevalence of EARR was calculated for each tooth. In order to identify potential risk factors, the following variables were assessed: sex, dental arch (maxillary or mandibular), treatment with or without extractions, treatment duration, root apex stage (open or closed), root shape, as well as overjet and overbite at treatment onset. Severity of resorption was scored as follows: 0-3 (none to mild EARR); 4 (severe EARR).

## STATISTICAL ANALYSIS

Results were formatted in a Microsoft Office Excel (version 2007, Microsoft Office Corporation) spreadsheet. Sample size calculation was performed, and the final sample was within the recommendations established for this study.

To assess intra and inter-rater reproducibility, kappa coefficient and chi-square test were used for comparison among groups. Level of probability was set at 5% (P < 0.05).

Both statistical tests and sample size calculation were performed with the aid of QuickCalcs GraphPad software (version 2013), available at www.graphpad.com/quickcalcs.

## RESULTS

Sample distribution is shown inTable 1. The means of treatment duration, overbite, overjet and changes between pre and post-treatment are demonstrated inTable 2. Overbite and overjet were measured by pre and post-treatment lateral cephalograms obtained in the same radiology center.

**Table 1 -:** Sample distribution.

Variable		n
Sex	Male	397
	Female	562
Extraction	Yes	413
	No	546
Treatment duration	≤ 3 years	174
	> 3 years	785
Angle’s classification	Class I	452
	Class II	428
	Class III	79

**Table 2 -:** Continuous variables.

Variable	Mean + SD	Minimum	Maximum
Initial overbite (mm)	2.37 ± 3.4	-4	9
Initial overjet (mm)	5.37 ± 4.14	-4	14
Change in overbite (mm)	1.86 ± 1.51	0	7
Change in overjet (mm)	2.57 ± 2.32	0	11
Treatment duration (years)	7.15 ± 3.97	1	14

According to the results shown inTable 3, maxillary central incisors had the highest percentage of severe EARR, followed by maxillary lateral incisors and mandibular lateral incisors. Out of 959 teeth, 28 (2.9%) had severe EARR.

**Table 3 -:** Prevalence of external apical root resorption (EARR) according to each tooth.

Tooth	Total		Degree of final resorption											
	n	(%)	Degree 0		Degree 1		Degree 2		Degree 3		Degree 0-3		Degree 4	
			n	(%)	n	(%)	n	(%)	n	(%)	n	(%)	n	(%)
11	121	100	24	(19.8)	19	(15.7)	55	(45.4)	15	(12.3)	113	(93.4)	8	(6.6)
12	118	100	22	(18.6)	16	(13.5)	56	(47.4)	19	(16.1)	113	(95.8)	5	(4.2)
21	120	100	26	(22.1)	20	(16.6)	51	(42.5)	15	(12.5)	112	(93.3)	8	(6.6)
22	118	100	26	(22.0)	18	(15.2)	49	(41.5)	20	(16.9)	113	(95.7)	5	(4.2)
31	120	100	43	(35.8)	41	(34.2)	30	(25.0)	6	(5.0)	120	100	0	(0.0)
32	120	100	53	(44.1)	33	(27.5)	30	(25.0)	3	(2.5)	119	(99.2)	1	(0.8)
41	121	100	49	(40.5)	40	(33.0)	27	(22.3)	5	(4.1)	121	100	0	(0.0)
42	121	100	60	(49.6)	29	(23.9)	27	(22.3)	4	(3.3)	120	(99.2)	1	(0.8)
Total	959	100	303	(31.6)	216	(22.5)	325	(33.9)	87	(9.0)	931	(97.1)	28	(2.9)

Table 4 shows the factors that could contribute to severe EARR. Anterior maxillary teeth, dental extraction for orthodontic purposes, treatment extended to more than three years, closed root apex at treatment onset and cases presenting overjet greater than or equal to 5 mm were statistically significant and, for this reason, were considered risk factors of EARR.

**Table 4 -:** Analysis of variables related to severe external root resorption (EARR).

Variable		Severe root resorption		Total (%)	χ^2^	P-value
		Absent n (%)	Present n (%)			
Sex	Male	389 (98.0)	8 (2.0)	397 (100)	1.95	0.162
	Female	542 (96.4)	20 (3.5)	562(100)		
Dental arch	Upper	451 (94.5)	26(5.4)	477 (100)	22.3	0.000
	Lower	480 (99.6)	2 (0.4)	482 (100)		
Extraction	Yes	389 (94.1)	24 (5.8)	413 (100)	21.3	0.000
	No	542 (99.2)	4 (0.7)	546 (100)		
Treatment duration	< 3 years	174 (100)	0 (0)	174 (100)	6.4	0.011
	> 3 years	757 (96.4)	28 (3.6)	785 (100)		
Apex	Open	264 (100)	0 (0)	264 (100)	10.9	0.000
	Closed	667 (96.6)	28 (4.0)	695 (100)		
Root shape*	Romboidal	325 (96.7)	11 (3.3)	336 (100)	0.97	0.324
	Triangular	342 (95.2)	17 (4.7)	359 (100)		
Overjet	< 5 mm	516 (98.7)	7 (1.3)	523 (100)	10.4	0.001
	≥ 5 mm	415 (95.2)	21 (4.8)	436 (100)		
Overbite	< 5 mm	693 (96.9)	22 (3.1)	715 (100)	0.24	0.624
	≥ 5 mm	238 (97.5)	6 (2.5)	244 (100)		

* The sum of root shapes T and R (695) corresponding to the number of teeth with closed apex.

Kappa coefficient revealed that agreement between the two measurement times was excellent (k = 0.84). Inter observer agreement was also excellent (k = 0.81).

## DISCUSSION

Periapical radiograph has been the examination most frequently used to evaluate EARR resulting from orthodontic treatment due to its higher accuracy compared to panoramic radiograph and better cost-benefit relationship compared to CT scans.^16^

In this study, apical dental alterations were classified according to the widely applicable and accepted index proposed by Malmgren et al,^17^ and modified by Levander et al.^9^ This method is predominantly used in root resorption studies performed after orthodontically induced tooth movement, and has the major advantage of not depending on standardization of initial radiographs.^13^^,^ 18 ^,^ 19 An important factor that must be considered in studies involving variables is the adequate review of the error of the method . The method used herein seems reliable, showing an excellent correlation between the two measurements. Intra and inter observer error of method was considered of little importance. These results validate the methods used to collect data in this research.

In the present investigation, the risk factors associated with severe EARR were teeth located in the anterior region of the maxillary arch, treatment involving extractions, treatment duration (over 3 years), overjet greater than or equal to 5 mm at treatment onset, and complete root formation (closed apex) also at treatment onset. It was not possible to relate the degree of resorption to root shape, the amount of overbite at treatment onset, or to patient's sex.

In agreement with the results of other researches,^1^^,^ 5 ^,^ 6 ^,^ 12 ^,^ 18 ^,^ 20 ^,^ 21 the present study found a low number of teeth with severe EARR (2.9%), while 97.1% showed no resorption or resorption classified as moderate, i.e., clinically accepted as part of the biological costs of orthodontic treatment. Marques et al^22^ analyzed 1,049 patients treated by means of the Edgewise technique alone. The authors found high percentages of severe resorption (14.5%). However, they reported difficulties in comparing the prevalence found in their research with the findings of other studies because their sample was larger than those found in the literature, which allowed the inclusion of more variables. Furthermore, they cited differences in methods and techniques as a factor that could help explain this discrepancy. Lim et al^23^ found differences in procedures used in routine clinical practice, such as the use of light forces and/or rest periods (discontinuous forces) every two to three months. Thus, groups of patients treated by different professionals, allied to the relatively recent advent of superelastic material enabling the use of light and progressive forces especially in the early stages of treatment,^4^^,^ 11 ^,^ 20 tend to show different final results.^5^^,^ 6 ^,^ 23

Anterior maxillary teeth proved more likely to present severe EARR than teeth located in the mandibular arch, which is in agreement with other studies.^5^^,^ 10 ^,^ 22 ^,^ 24 ^,^ 25 ^,^ 26 Previous research on intrusion and retraction movements of anterior teeth with lingual root torque,^2^^,^ 12 required to reduce overjet^7^ and to close extraction spaces, might support this finding. According to Martins et al*,* 19 patients treated with intrusion mechanics combined with anterior retraction had statistically greater maxillary incisor root resorption than those treated with anterior retraction without intrusion. This finding is probably related to greater tooth movement necessary to close extraction spaces,^8^^,^ 27 specially when associated with intrusive mechanics^25^ and torque movement,^2^^,^ 10 ^,^ 12 which overburdens the dental apex. In addition, proximity between the roots of maxillary central incisors and the cortical bone of the socket, the incisive canal and the alveolar bone on the buccal surface, combined with the type of movement may explain the higher incidence of severe EARR in these teeth.^24^ On the other hand, if the extraction space is used to relieve crowding,^28^ which is usual in the mandibular arch, incisors might not be submitted to major retractions. This could explain the discrepancy between maxillary and mandibular teeth in this study.

The present investigation found that treatment duration was significantly correlated with severe EARR. Extended treatment duration is cited as a risk factor in the development of severe EARR,^5^^,^ 6 ^,^ 10 ^,^ 26 although some authors do not agree with this finding.^1^^,^ 8 ^,^ 13 ^,^ 19 ^,^ 21 Confounding factors, such as more difficult treatment plans, appointment intervals and lack of patient's cooperation, can increase treatment time and also be related to EARR.^26^ Moreover, longer treatment time might reflect more severe malocclusion and the need for different treatment mechanics, thereby resulting in extended period of time for treatment finishing. For example, by assessing the influence of metal and ceramic brackets on root resorption, some authors reported a higher incidence of EARR in patients treated with ceramic brackets. According to these authors, treatment with ceramic brackets lasts longer, which may explain these findings.^29^ Harris and Baker^30^ stated that there is a threshold time at which the dynamic process is overwhelmed and significant resorption takes place. Therefore, it can be hypothesized that continuous stimulation of the root leads to increased root resorption, and accumulation of surface root resorption over a long period of time can lead to the onset of severe EARR.^24^

We did not assess the association between intermaxillary elastics and EARR in this study. However, several authors have related the use of elastics and EARR,^8^^,^ 24 ^,^ 25 while others have not found this association in their studies.^6^ In our sample, all patients used elastics for treatment finishing. Those who showed less cooperation usually had treatment time and the use of elastics increased. It seems reasonable to assume that long-term jiggling forces caused by intermittent use of elastics can be a contributing factor in the prevalence of EARR.^24^

Most studies have found an association between orthodontic treatment with extraction and the presence of severe EARR.^5^^,^ 6 ^,^ 24 ^,^ 27 In the present study, cases with extraction presented significantly more severe EARR than those treated without extractions. Increased movement and retraction of the apex of incisors are necessary to close extraction spaces. Additionally, extraction cases usually require longer treatment time for orthodontic treatment finishing. Thus, it could be assumed that tooth extraction can increase the amount of movement and the duration of treatment, thereby playing an important role as a risk factor.

With respect to overjet, significant association between its magnitude and the presence of severe EARR was observed, which is in agreement with other researches.^3^^,^ 5 ^,^ 6 ^,^ 8 ^,^ 28 Brin *et al* 3 reported similar association in incisor retraction used to reduce overjet during fixed-appliance treatment. Nevertheless, this type of tooth movement was reduced in patients who underwent early therapy to reduce Class II malocclusion (e.g., headgear and/or functional appliances as a first phase of treatment). The authors stated that early growth modification, which reduces the severity of overjet in Class II malocclusions, might play an important role in reducing the likelihood of severe EARR.

It was found that teeth with complete root formation at treatment onset are more likely to develop severe EARR, which is in agreement with other researches.^28^^,^ 29 Teeth with incomplete root formation at orthodontic treatment onset continue to develop their roots during therapy.^29^ In adults, the periodontal ligament becomes less vascularized, aplastic and narrow; the bone becomes denser, avascular and aplastic; and the cementum wider.^28^ These physiological changes could explain the higher susceptibility to severe EARR found in this study.

In contrast to other studies, our study revealed no correlation between patient's sex, root shape, the amount of overbite at treatment onset and the amount of severe EARR.Table 2 shows that our sample presented lower mean values of overbite than those found for overjet, for values measured before treatment and the reduction values of these variables. This may explain the poor relationship between overbite and EARR found in our study.

The results of this study suggest that care must be taken in orthodontic treatment with extraction, in which great retraction of maxillary incisors is planned; treatment that exceeds three years; and specially treatment involving anterior maxillary teeth with completely formed apex at orthodontic treatment onset. Considering that severity of malocclusion, rather than its type (e.g. Angle's classification),^8^ is a determining factor in the amount and type of tooth movement as well as in the orthodontic mechanics used and the duration of orthodontic treatment, it can be assumed that EARR has a multifactorial cause, regardless of the sagittal characteristics of malocclusion.

## CONCLUSION

The prevalence of severe EARR resulting from orthodontic treatment was considered low in this study (2.9%). Risk factors involved were as follows: treatment with extraction, anterior maxillary teeth, overjet greater than or equal to 5 mm at treatment onset, prolonged therapy and teeth with complete root formation at treatment onset; all of which suggest that EARR is a multifactorial phenomenon.
